# The Influence of a Genetic Variant in *CCDC78* on *LMNA*-Associated Skeletal Muscle Disease

**DOI:** 10.3390/ijms25094930

**Published:** 2024-04-30

**Authors:** Nathaniel P. Mohar, Efrem M. Cox, Emily Adelizzi, Steven A. Moore, Katherine D. Mathews, Benjamin W. Darbro, Lori L. Wallrath

**Affiliations:** 1 Interdisciplinary Graduate Program in Genetics, University of Iowa, Iowa City, IA 52242, USA; nathaniel-mohar@uiowa.edu (N.P.M.); emily-adelizzi@uiowa.edu (E.A.); 2 Department of Biochemistry and Molecular Biology, Carver College of Medicine, University of Iowa, Iowa City, IA 52242, USA; 3 Department of Pathology, Carver College of Medicine, University of Iowa, Iowa City, IA 52242, USAsteven-moore@uiowa.edu (S.A.M.); 4 Department of Neurosurgery, UNLV School of Medicine, Las Vegas, NV 89106, USA; 5 Department of Anatomy and Cell Biology, Carver College of Medicine, University of Iowa, Iowa City, IA 52242, USA; 6 Department of Pediatrics, Carver College of Medicine, University of Iowa, Iowa City, IA 52242, USA; katherine-mathews@uiowa.edu

**Keywords:** core myopathy, genetic modifiers, lamins, laminopathy, limb–girdle muscular dystrophy

## Abstract

Mutations in the *LMNA* gene-encoding A-type lamins can cause Limb–Girdle muscular dystrophy Type 1B (LGMD1B). This disease presents with weakness and wasting of the proximal skeletal muscles and has a variable age of onset and disease severity. This variability has been attributed to genetic background differences among individuals; however, such variants have not been well characterized. To identify such variants, we investigated a multigeneration family in which affected individuals are diagnosed with LGMD1B. The primary genetic cause of LGMD1B in this family is a dominant mutation that activates a cryptic splice site, leading to a five-nucleotide deletion in the mature mRNA. This results in a frame shift and a premature stop in translation. Skeletal muscle biopsies from the family members showed dystrophic features of variable severity, with the muscle fibers of some family members possessing cores, regions of sarcomeric disruption, and a paucity of mitochondria, not commonly associated with LGMD1B. Using whole genome sequencing (WGS), we identified 21 DNA sequence variants that segregate with the family members possessing more profound dystrophic features and muscle cores. These include a relatively common variant in *coiled-coil domain containing protein 78* (*CCDC78*). This variant was given priority because another mutation in *CCDC78* causes autosomal dominant centronuclear myopathy-4, which causes cores in addition to centrally positioned nuclei. Therefore, we analyzed muscle biopsies from family members and discovered that those with both the *LMNA* mutation and the *CCDC78* variant contain muscle cores that accumulated both CCDC78 and RyR1. Muscle cores containing mislocalized CCDC78 and RyR1 were absent in the less profoundly affected family members possessing only the *LMNA* mutation. Taken together, our findings suggest that a relatively common variant in *CCDC78* can impart profound muscle pathology in combination with a *LMNA* mutation and accounts for variability in skeletal muscle disease phenotypes.

## 1. Introduction

The nuclear lamina is a dense network of intermediate filaments and membrane associated proteins that line the surface of the inner nuclear membrane in eukaryotic cells [1,2,3]. Functions of the nuclear lamina include interacting with the Linker of Nucleus and Cytoskeleton (LINC) complex involved in sensing mechanical stress, making connections with chromatin to regulate gene expression, and enabling nuclear transport by anchoring nuclear pore complexes [4,5,6]. A major component of the nuclear lamina are lamins, which are type V intermediate filaments [1]. Lamins are composed of a globular head domain, an alpha helical rod possessing several linkers, and a tail domain possessing both a nuclear localization sequence (NLS) and an Ig-like fold domain [2,3]. Lamins dimerize through the rod domain; the dimers then form head-to-tail filaments and assemble into higher order structures [1,7]. Lamins are classified into A- and B-types [5,8,9,10]. The B-type lamins, B1 and B2, are encoded by the human genes *LMNB1* and *LMNB2,* respectively, and are constitutively expressed [9]. The human A-type lamins, A and C, are encoded by the *LMNA* gene, produced via alternative splicing, and expressed upon differentiation [8]. Lamin C is directly translated from *LMNA* mRNA; however, lamin A is translated as prelamin A and requires additional post-translational processing to become mature lamin A [5,8].

Mutations in the genes encoding lamins result in a diverse class of diseases known as laminopathies [11,12,13]. The majority of laminopathies are caused by *LMNA* mutations [12]. At least 500 pathogenic variants in *LMNA* have been reported as causal of at least 16 distinct phenotypes, most of which are inherited in an autosomal dominant fashion [11,13,14,15]. These phenotypes include the early-onset aging syndrome Hutchinson–Gilford Progeria Syndrome (HGPS), striated muscle diseases, lipodystrophy, and peripheral neuropathy [11,13,16]. This genetic heterogeneity is not defined by the protein domain affected, as mutations that cause each disease are dispersed throughout the gene and affect different protein domains [17,18,19], with the exception of a G608G splice site mutation being the primary cause of HGPS [20,21,22]. 

The *LMNA*-associated striated muscle diseases include dilated cardiomyopathy (DCM) with conduction defects, Emery–Dreifuss muscular dystrophy Type 2 (EDMD2), lamin-associated congenital muscular dystrophy (L-CMD), and Limb–Girdle muscular dystrophy Type 1B (LGMD1B) [16,23,24,25,26]. According to the Muscular Dystrophy Association, LGMD1B is the most common dominant form of LGMD and accounts for 5-10% of total LGMD cases [25,27]. LGMD is characterized by weakness and atrophy of the proximal muscles around the shoulder and pelvic girdles [28,29]. LGMD1B frequently co-occurs with DCM and arrhythmias, which ultimately can reduce the life expectancy of affected individuals [25,28,30].

A hallmark of striated muscle laminopathies, including LGMD1B, is phenotypic variability [16]. The same *LMNA* mutation can give rise to clinically distinct phenotypes in different individuals, even among closely related family members [14,17,31,32,33]. Variability exists with age at onset of symptoms, rate of disease progression, and disease severity [32,34,35,36,37,38,39,40,41]. This variability has been attributed to genetic background; however, little progress has been made in identifying genetic background variants that influence the phenotypes of *LMNA*-associated muscular dystrophy (*LMNA*-MD) patients [16,42,43,44,45]. Mining whole genome sequencing (WGS) data for variants that co-segregate with a disease trait can identify variants that contribute to phenotypic variability [46,47,48,49].

Here, we describe a four-generation family in which members present with either dominant LGMD, DCM, or both. DNA sequence analysis revealed that affected family members possess a mutation in *LMNA* that alters *LMNA* pre-mRNA splicing. Along with this variability in diagnosis, there is variability in the skeletal muscle pathology in this family. Some individuals diagnosed with LGMD1B also possess skeletal muscle cores, a histopathologic phenotype not commonly seen in cases of *LMNA*-MD or LGMD [25,27,28,50,51]. These individuals have a more pronounced skeletal muscle pathology and are more severely affected than their siblings who lack cores. Thus, the phenotypic variability and the presence of muscle cores was hypothesized to be due to genetic background differences among the family members. To identify such background differences, we mined WGS of the family members and identified a relatively common variant in *CCDC78*, a known core myopathy gene [50,51,52], that co-segregates with muscle cores. We show that this variant, in combination with the *LMNA* mutation, causes profound skeletal muscle disease and core myopathy. Thus, a rare *LMNA* disease-causing mutation appears to sensitize the muscle to effects of a common *CCDC78* variant, leading to multiple muscle diseases and a more severe phenotype than the *LMNA* mutation alone.

## 2. Results

### 2.1. Clinical Features of a Multigenerational Family with Cardiac and Skeletal Muscle Defects

**Figure 1:**
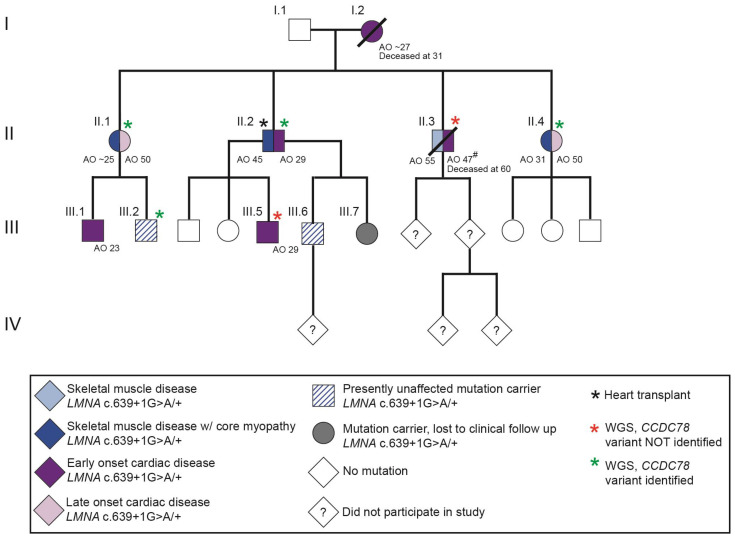
The pedigree of a family with dominant skeletal muscle and cardiac disease is shown. AO, age at onset (years) for skeletal muscle disease on the left and cardiac disease on the right; #, LVEF of the individual reduced to 35% upon initial examination and age of onset is presumed to be earlier than reported.

In this study, we investigate the genetic basis of disease in a four-generation family with adult-onset LGMD and/or cardiomyopathy (Figure 1). Clinical features of all affected family members are summarized in Table 1. Medical histories are briefly described below.

**Figure 2:**
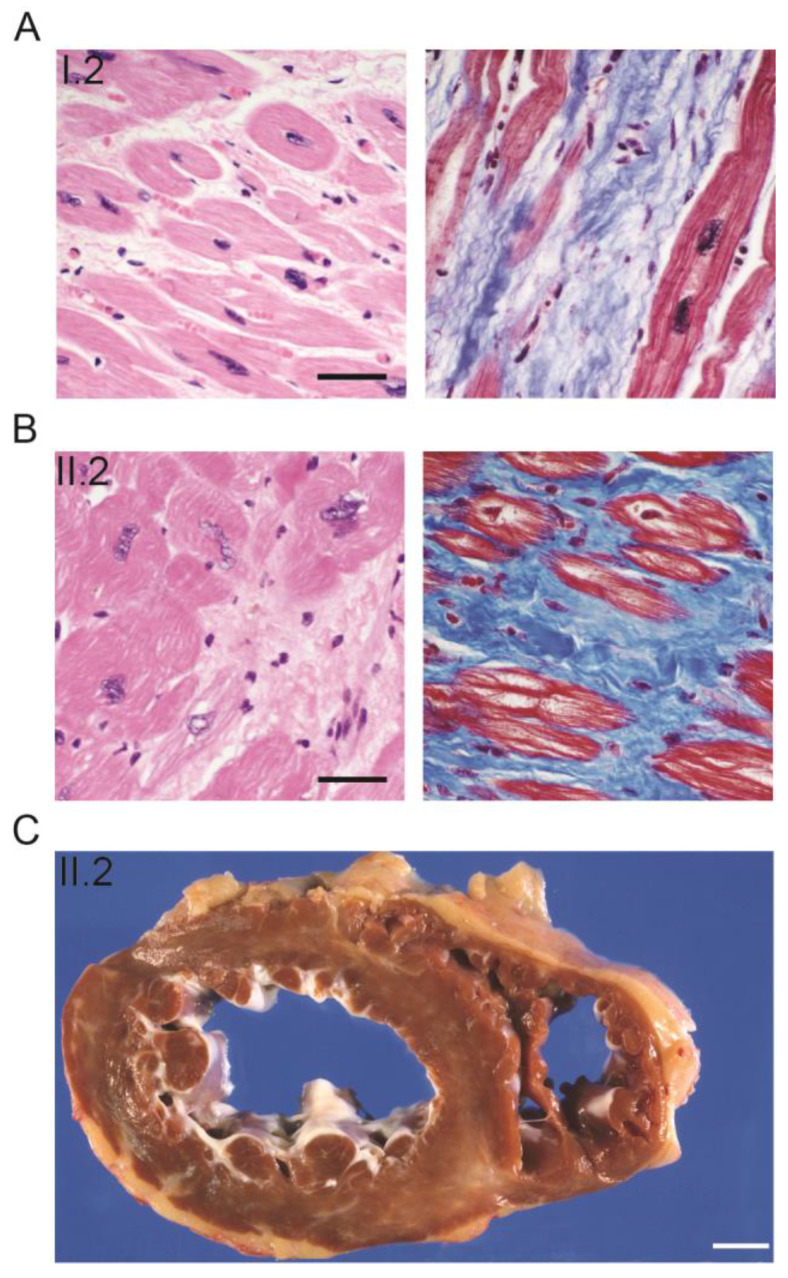
Cardiac pathology of two family members with heart failure. (**A**) Photomicrographs of cardiac tissue from the autopsy of I.2 stained with hematoxylin and eosin (**left**) and Masson trichrome (**right**); illustrates myocardial fibrosis, hypertrophic cardiomyocytes, and large, pleomorphic myocyte nuclei. (**B**) Photomicrographs of tissue from the explanted heart from individual II.2; illustrates features of chronic heart failure like individual I.2. Scale bar for all photomicrographs = 50 µm. (**C**) Gross photograph of the explanted heart from individual II.2; depicts ventricular dilation and scarring of the left ventricular wall and interventricular septum (grey–white regions within the dark brown cardiac muscle). Scale bar = 1 cm.

Individual I.2 died of dilated cardiomyopathy confirmed with postmortem examination at age 31 (Figure 2A). A postmortem examination of skeletal muscle was not conducted.

Individual II.1 recalls exercise intolerance in grade school but did not recognize muscle weakness until her mid-twenties when she had difficulty arising from chairs and climbing stairs. She had an implantable cardioverter–defibrillator (ICD) placed at age 50 for abnormal ECG and later developed atrial and ventricular arrhythmias with preserved left ventricular ejection fraction (LVEF). She began using a wheelchair full-time at age 59. 

Individual II.2 presented at 29 years old with new-onset near-syncope. Motor function was normal, although an elevated serum creatine kinase (CK) prompted a muscle biopsy. He was followed at an outside hospital until he returned to University of Iowa Hospitals and Clinics (UIHC) at the age of 41. Neuromuscular evaluation as part of transplant discussion due to heart failure detected subtle gait abnormality but he reported no clinical muscle weakness. He underwent ventricular assist device (VAD) followed by heart transplant for heart failure and cardiac arrhythmia at age 43 years. Explanted heart pathology was consistent with the diagnosis of dilated cardiomyopathy (DCM) (Figure 2B,C). He later developed symptomatic muscle weakness (e.g., trouble climbing stairs) by age 45 years. He was able to walk up to a block at age 60 but used power mobility for greater distances. 

Individual II.3 had his first abnormal ECG and echocardiogram found on routine cardiac monitoring for an unrelated procedure at age 47 years, and LVEF was decreased to 35–40% upon initial evaluation. He had no cardiac or neuromuscular symptoms. He underwent ICD placement at age 48 years. He started to experience weakness (trouble climbing stairs) at age 55 years. He remained ambulatory with near-normal strength until his health was adversely impacted by unrelated medical illnesses and he died at age 60. 

Individual II.4 developed heart failure associated with macrodantin use at ~30 years. Cardiac function recovered, but following hospitalization, she developed hip girdle weakness. At age 50 years, she had normal cardiac function, but an electrophysiology study showed inducible atrial fibrillation, resulting in a pacemaker placement at age 58 years. She lost ambulation around age 60 years, at least in part due to unrelated health problems. 

At age 23, Individual III.1 had a normal neurologic examination but reported episodes of unprovoked tachycardia and palpitations. He had normal cardiac function but had paroxysmal atrial fibrillation. An electrophysiology study was reassuring, and he was started on a beta-blocker. When he was 28 years old, he again experienced cardiac palpitations, which were confirmed to be runs of nonsustained ventricular tachycardia. Cardiac MRI with and without contrast was normal and he had an ICD placed. He has no symptoms of skeletal muscle weakness. 

Individual III.2 originally presented with mild lower extremity weakness, difficulty running, and easy falling in childhood, but this weakness resolved. He was normal upon exam in adulthood, although he is currently below the age of symptomatic weakness onset of the other males in the family. There is possible significance of male sex in skeletal muscle disease onset in this family, as the two males in generation II retained normal strength much longer than the two females. He is assumed to be presymptomatic.

Individual III.5 has never experienced skeletal muscle weakness. A routine echocardiogram showed decreased function at age 29, and he was managed medically. At 30 years old, cardiac MRI showed LVEF of 35% and an area of linear mid-myocardial delayed enhancement. He remained asymptomatic and underwent preventative ICD placement.

Individual III.6 has never experienced either skeletal muscle weakness or cardiac symptoms but is seen due to the family’s history. A cardiac MRI at age 38 showed normal heart function and no late gadolinium enhancement. He is assumed to be presymptomatic.

### 2.2. Skeletal Muscle Pathology of the Four Generation II Siblings Contains Dystrophic Features

**Figure 3:**
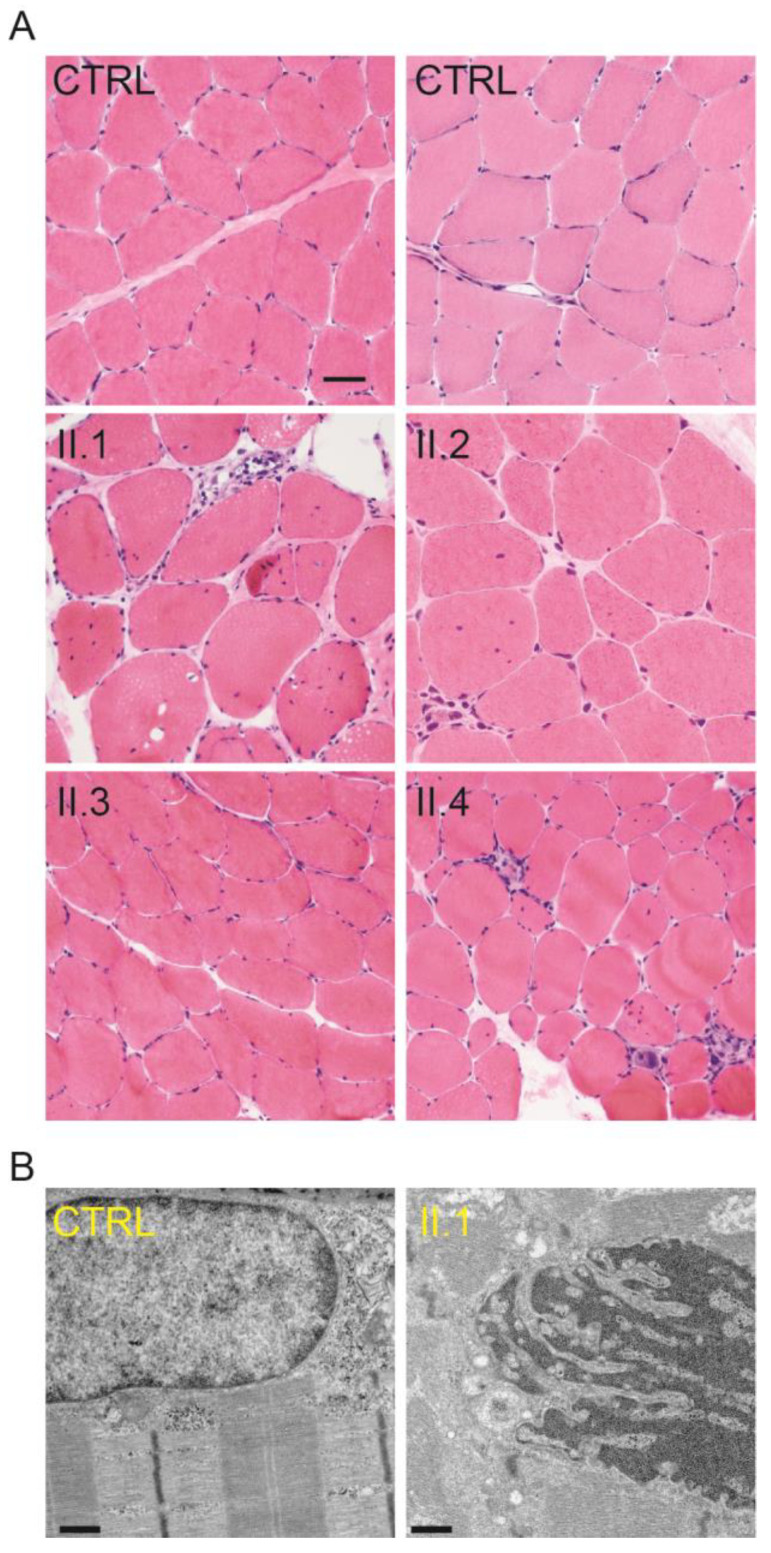
Skeletal muscle tissue from individuals in generation II exhibit dystrophic features. (**A**) Cryosections of muscle biopsies from individuals II.1, II.2, and II.4 stained with H&E show abnormal variation in fiber size and increased internal nuclei relative to the “no diagnostic abnormality” controls (CTRL), as well as scattered muscle fibers undergoing necrosis or regeneration. Note that the cryosection of muscle biopsy from individual II.3 shows milder dystrophic features. Scale bar for all photomicrographs = 50 µm. (**B**) Electron microscopy of muscle biopsy tissue from individual II.1 depicts nuclear invaginations that are not present in muscle from a control (CTRL). Scale bar = 1 µm.

**Figure 4:**
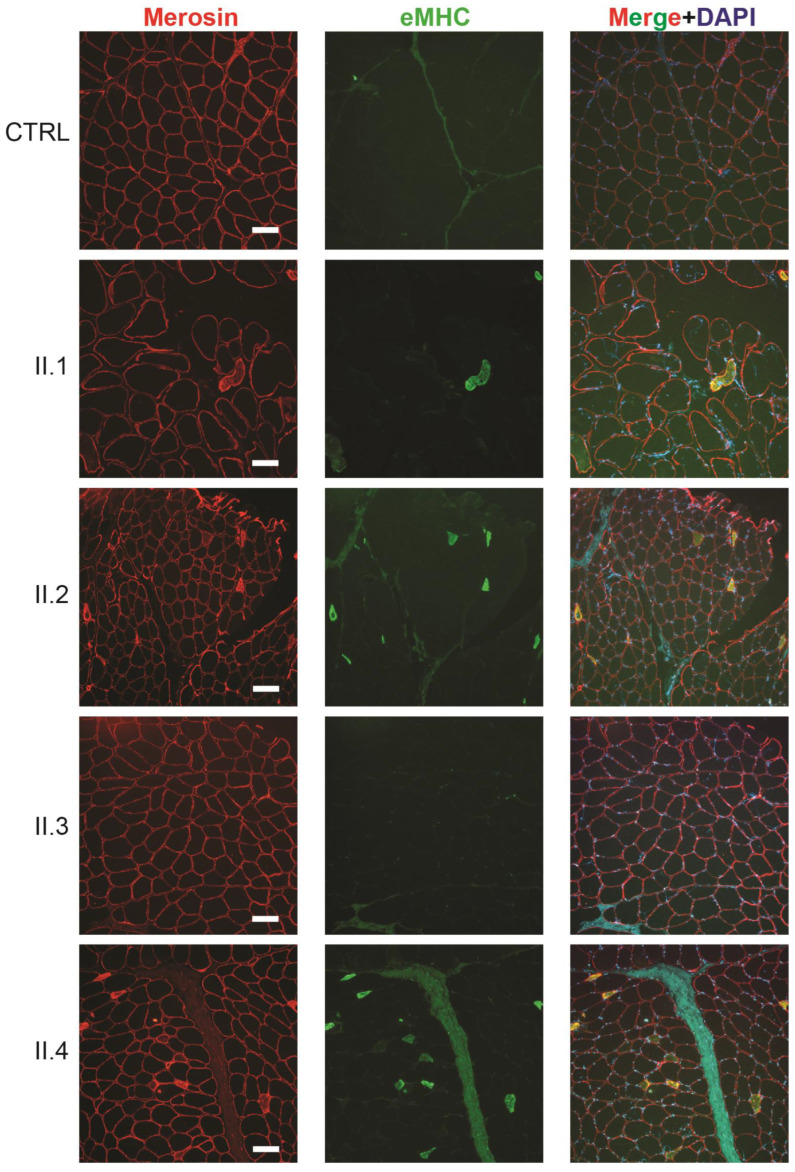
Skeletal muscle tissue from individual II.3 shows fewer regenerating fibers than muscle tissue from their siblings. Cryosections of muscle biopsy tissue from generation II family members stained with antibodies against merosin and embryonic myosin heavy chain (eMHC) show regenerating fibers present in individuals II.1, II.2, and II.4 that are not present in individual II.3 or from a control (CTRL). Scale bar for all photomicrographs = 50 µm.

Skeletal muscle biopsies from the four generation II siblings showed dystrophic features including internalized nuclei and fiber size variation that were not present in controls (Figure 3A). These dystrophic features were more pronounced in muscle from individuals II.1, II.2, and II.4 than they were in individual II.3. Electron microscopy revealed nuclear invaginations that were not present in a control, suggesting the disease may be caused by a mutation in a gene encoding a nuclear envelope protein (Figure 3B). Further analysis of the skeletal muscle biopsy tissue included a stain for embryonic myosin heavy chain (eMHC) to identify regenerating fibers (Table S1). Regenerating fibers were clearly seen in individuals II.1, II.2, and II.4, but largely absent in individual II.3 and an unrelated control (Figure 4). This finding is consistent with individual II.3 appearing less dystrophic on the H&E stain and having a milder skeletal muscle phenotype than his siblings.

### 2.3. A Novel LMNA Variant Was Identified as Causal of These Phenotypes

Clinical diagnostic sequence analyses of genomic DNA from this family revealed a variant in the *LMNA* gene (OMIM#150330) as a potential cause of the disease phenotypes. The sequence of the *LMNA* gene in these individuals was evaluated via traditional Sanger sequencing of genomic DNA extracted from peripheral blood leukocytes. The 12 coding exons and the flanking intronic boundaries were amplified with PCR and sequenced in both directions. The patient DNA sequence was compared to the *LMNA* reference sequence (NM_170707). A heterozygous variant (c.639+1G>A) was found in the *LMNA* gene of all affected family members of generation II, the affected individual in generation I, and five members of the third generation (III.1, III.2, III.5, III.6, and III.7), resulting in a clinical diagnosis of LGMD1B [26,28,53].

**Figure 5:**
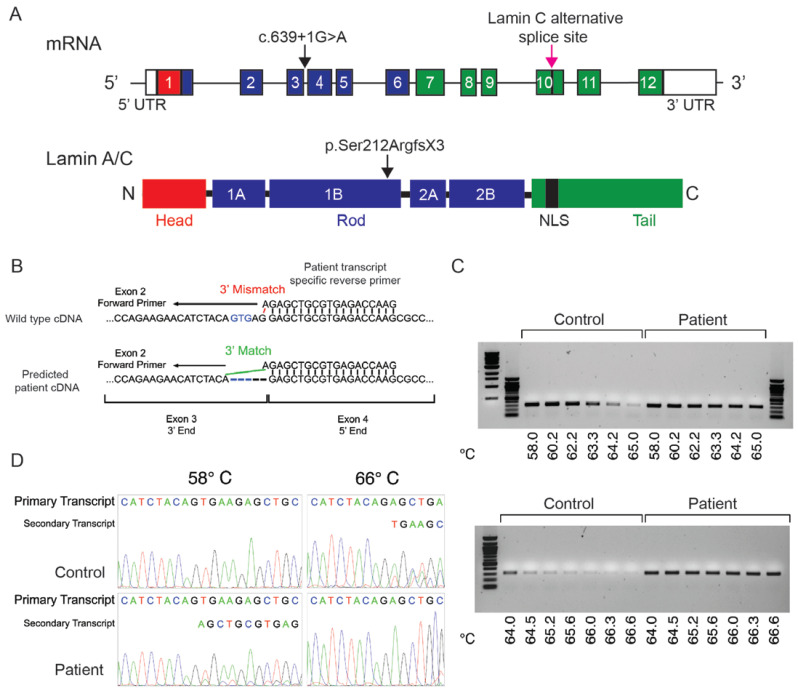
PCR was used to validate the effect of the *LMNA* mutation on pre-mRNA splicing. (**A**) A linear diagram of *LMNA* gene with the location of the altered nucleotide shown by the arrow. A linear diagram of the lamin A/C protein with the protein domains and the location of the resulting amino acid substitution indicated by the arrow. (**B**) PCR primers were designed to detect the presence of a predicted spliced mRNA lacking the five terminal nucleotides of exon three (predicted with in silico analysis) in fibroblasts of an affected family member. A PCR primer was designed to differentially amplify this mutant transcript and wild-type transcript. (**C**) RT-PCR analysis of total mRNA extracted from fibroblasts of a control and an affected family member using the mutant transcript specific primer. The annealing temperature of the PCR reaction was increased from 58 °C to nearly 67 °C resulting in differential amplification of the wild-type transcript (highest efficiency at lower temperatures) and mutant transcript (high efficiency at both lower and higher temperatures). (**D**) Sanger sequencing of DNA amplification products from RT-PCR analysis at 58 °C and 66.6 °C is shown. Both the primary (most abundant) and secondary (less abundant) transcript sequences are highlighted. Sequencing using the forward primer revealed that the predicted mutant transcript is present and can be enriched at higher annealing temperatures. The results of the sequencing analysis provide evidence that the predicted mutant transcript is produced at significant levels in the fibroblasts from an affected family member, and that it may also be produced in the control fibroblasts at a very low level.

This *LMNA* mutation is absent in population DNA sequence databases such as gnomAD, suggesting that it is extremely rare. However, this variant has been associated with cardiomyopathy in a literature report [54] and is listed in ClinVar (variant ID 633290) and LOVD (Leiden Open Variation Database), with the LOVD entry representing a previous report of this family [55]. The c.639+1G>A change affects the known in vivo utilized 5′ donor site of intron 3, leading us to hypothesize that the mutation might cause aberrant pre-mRNA splicing [56]. In silico modeling predicted that the alternative splicing caused by this DNA sequence change result in a loss of five nucleotides from the spliced mRNA transcript, a shift in the reading frame, and generation of a premature stop codon after the third codon of the spliced exon four (c.639+1G>A, r.635_639del, [p.Ser212ArgfsX3]) (Figure 5A).

Two experimental approaches were used to test the results of the in silico analysis. The first was a full transcript analysis in which all twelve exons of *LMNA* were interrogated with semi-quantitative RT-PCR performed on total RNA extracted from cultured fibroblasts from a healthy control individual and individual II.2. Overlapping amplicons spanning all twelve exons of *LMNA*, including one primer pair designed to amplify *LMNA* transcript isoform 2 (NM_005572) that gives rise to lamin C proteins, showed no difference in either size or quantity of transcript between control and affected fibroblasts (Figure S1). Furthermore, no abnormally sized amplicons were detected in any of the amplification reactions (Figure S1). Primer sequences used for this analysis are provided in Table S2.

This analysis could not rule out utilization of the cryptic splice site five base pairs upstream as predicted with the in silico analysis. Agarose gel electrophoresis cannot resolve two amplicons that differ in size by only five base pairs. While polyacrylamide gel electrophoresis could resolve this difference, the mutant transcript is unlikely to be detectable on any form of gel if it is degraded by nonsense mediated decay. However, detection of a mutant transcript, even if partially degraded by nonsense mediated decay, can be detected with allele-specific PCR amplification. We designed a PCR primer that would be specific to the mutant exon 3:exon 4 junction in the reverse-transcribed cDNA (Figure 5B). This junction would be missing five base pairs if the cryptic splice site was utilized. Using a nonspecific forward primer coupled with our mutant-specific reverse primer, we performed the PCR amplification reactions at increasingly stringent annealing temperatures, thus increasing the specificity of the mutant primer for the mutant transcript and reducing amplification of wild-type cDNA (Figure 5C). The gradient annealing temperature amplification clearly demonstrated a barely detectable product in the control fibroblasts at higher annealing temperatures but continued robust amplification of a product from the cDNA of the affected fibroblasts. 

We then sequenced the amplification products produced with PCR (Figure 5D). Even though the wild-type transcript was preferentially amplified from both the control and affected fibroblasts at lower annealing temperatures, the predicted mutant transcript was detected in the affected cells, but not the control cells. Sequencing the products produced at higher annealing temperatures confirmed that the sole product in the affected fibroblasts was the predicted mutant transcript and that this mutant transcript was produced at very low levels in the control cells. These results confirmed the in silico prediction that cells from affected individuals carrying the c.639+1 G>A mutation in *LMNA* possess both a wild-type transcript and an aberrantly processed transcript. This aberrant transcript utilizes a cryptic donor splice site five base pairs upstream of the authentic intron three 5′ donor splice site.

### 2.4. Muscle Cores Are Present in a Subset of the LGMD1B-Affected Individuals

**Figure 6:**
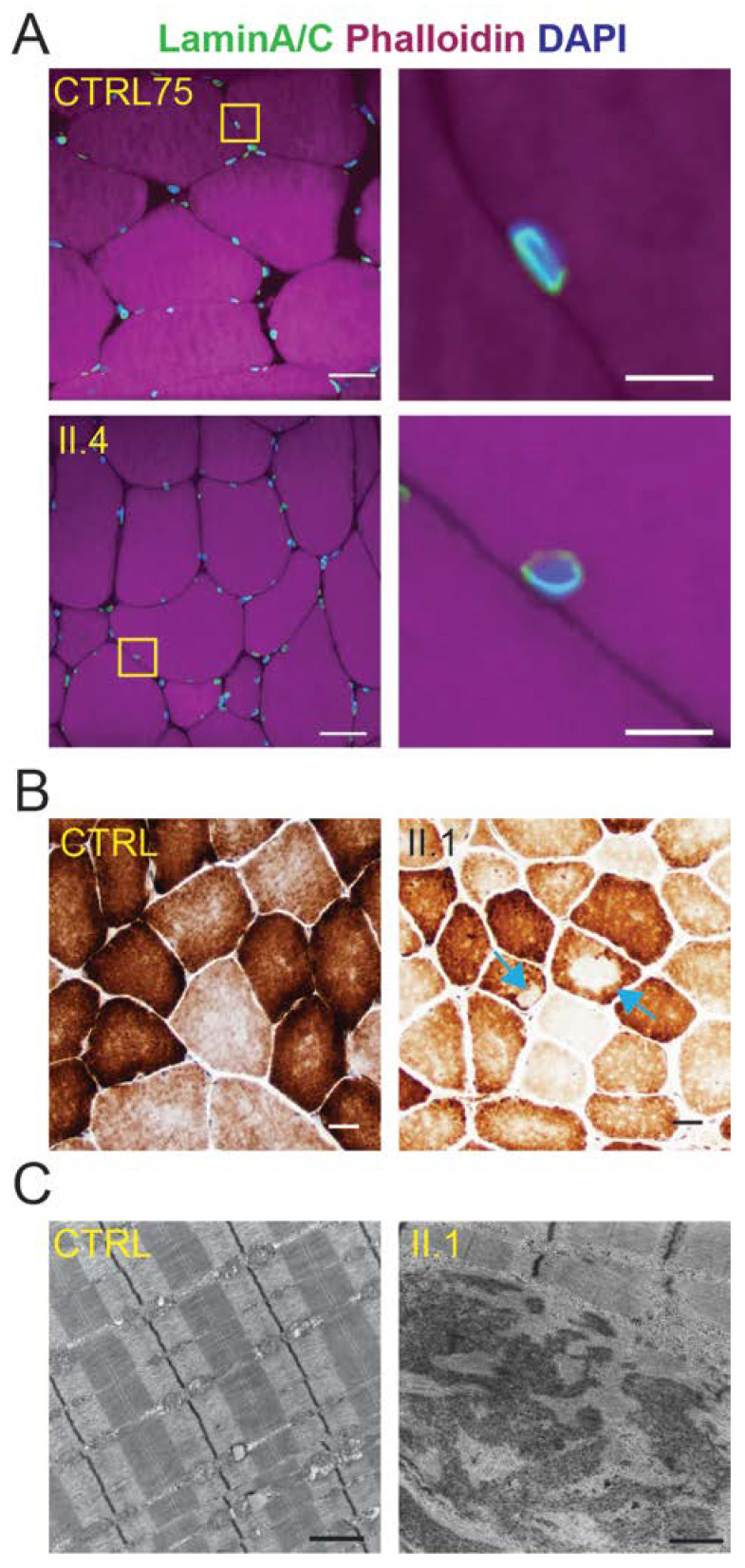
Skeletal muscles from affected family members show the anticipated localization of nuclear envelope proteins; however, profoundly affected individuals have muscle cores. (**A**) Skeletal muscle biopsies from patient II.4 and an unrelated control (CTRL75) stained with an antibody against lamin A/C showed the anticipated localization at the nuclear periphery. The enlarged region of the full images is indicated by the yellow box. Scale bar on full images = 30 µm, Zoomed images = 5 µm. (**B**) Individuals profoundly affected with skeletal muscle disease such as II.1 show cytoplasmic regions of pallor with cytochrome C oxidase (COX)-staining, indicated by the blue arrows. The lack of staining indicates areas devoid of mitochondria which are known as muscle cores. Scale bar = 50 µm. (**C**) Electron microscopy of skeletal muscle biopsy tissue from individual II.1 depicts the regional disruption of skeletal muscle sarcomeric ultrastructure, including Z banding, which is indicative of muscle cores. Such disorganization was not observed in muscle from a control individual (CTRL). Scale bar = 1 µm.

To determine the impact of the *LMNA* mutation on nuclear envelope protein localization, immunostaining with antibodies to lamin A/C, emerin, and FG-repeat-containing nuclear pore proteins (FG-NUPs) were performed (Table S1). Despite the presence of the *LMNA* mutation, the localization of lamin A/C was not altered in a patient compared to control (Figure 6A). Consistent with normal lamin A/C localization, the staining with antibodies to emerin and FG-NUPs showed the anticipated nuclear envelope localization (Figure S2A,B).

To further investigate cellular changes associated with the skeletal muscle disease, histochemical staining for cytochrome C oxidase (COX) was performed. Skeletal muscle from individual II.1 showed an area of cytoplasmic pallor on the COX stain that was abnormal relative to controls (Figure 6B). In total, individuals II.1, II.2, and II.4 were found to have this abnormal phenotype while II.3 does not, consistent with the fact that these three individuals had a similar muscle disease presentation while individual II.3 had a relatively mild phenotype. This cytoplasmic pallor (COX negative staining) is consistent with the presence of muscle cores, regions of individual muscle fibers with sarcomeric disruption, and a paucity of mitochondria [50,57,58]. Electron microscopic evaluation of muscle biopsy tissue from individual II.1 further supported the presence of muscle cores, showing a regional loss of the characteristic Z banding pattern accompanied by disruption of the sarcomeric ultrastructure (Figure 6C) [59,60]. Cytoplasmic areas of COX negative staining have been observed for several core myopathies [51,52,61]; however, the presence of muscle cores in *LMNA*-MD is atypical. Moreover, muscle cores are also not typically part of the pathology of LGMD [28], making this a unique finding within members of this family. 

### 2.5. A Relatively Common Variant in CCDC78 Was Identified in Family Members with Muscle Cores

The variability in skeletal muscle phenotypes in this family and the atypical presence of muscle cores in profoundly affected family members suggest the influence of genetic background. To identify background genetic variants that contribute to this variability, we performed whole genome sequencing (WGS) on six individuals within the family, individuals II.1-4, III.2, and III.5. All sequenced individuals have the *LMNA* mutation, confirming the prior clinical laboratory test results. To identify variants that segregated with the muscle core phenotype, we compared WGS data from individuals II.1, II.2, and II.4 to that of individuals II.3 and III.5. Individuals II.1, II.2, and II.4 are profoundly affected by muscle disease with cores, while individual II.3 is less profoundly affected and does not have muscle cores and individual III.5 has cardiac disease with no evidence of skeletal muscle involvement. All single nucleotide and small insertion/deletion variants were annotated and filtered for quality control, minor allele frequency <5%, and predicted loss-of-function or missense mutations. The analysis revealed 21 variants with the proper segregation pattern that were rank ordered according to conservation, gene constraint metrics, in silico predictors of pathogenicity, relevant tissue expression, and annotated gene association with the reported phenotype [62,63,64,65,66,67,68,69,70,71,72,73]. A list of these 21 variants in rank order is provided in Table 2.

**Figure 7:**
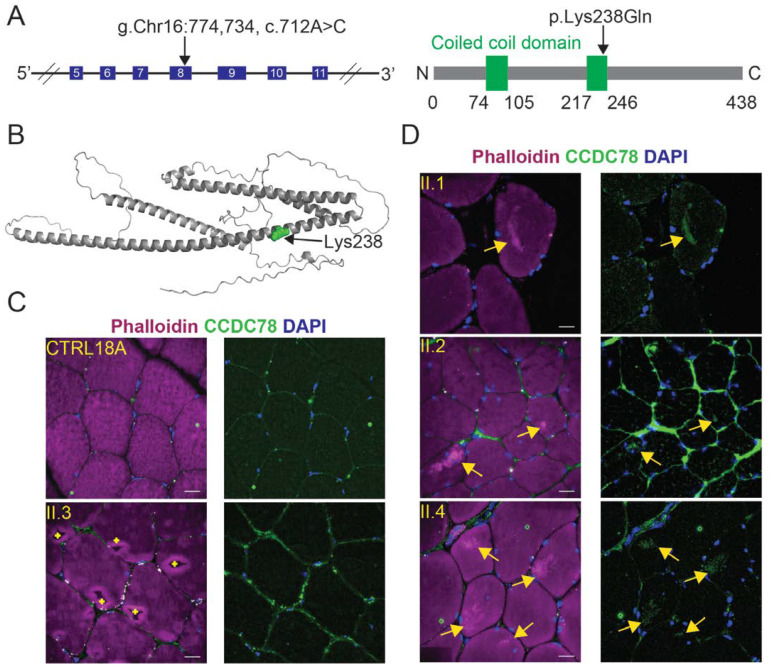
A *CCDC78* variant causes accumulation of CCDC78 in muscle cores. (**A**) A diagram of a portion of the *CCDC78* gene is shown at the left. The coding exons (numbered boxes) and the location of the nucleotide affected by the sequence variant are indicated. A linear diagram of the CCDC78 protein structure with the location of the predicted amino acid substitution indicated at the right. (**B**) The AlphaFold predicted structure of CCDC78 with lysine 238 indicated in green, which is changed to glutamine by the sequence variant. (**C**) Skeletal muscle biopsy tissue from an unrelated control (CTRL 18A, top) and a family member (II.3) with the *LMNA* variant but lacking the *CCDC78* variant shows CCDC78 localization to the periphery of muscle fibers and uniform phalloidin staining throughout the cytoplasm. Scale bars = 30 µm. + = evidence of ice-crystal artifacts from tissue preservation (**D**) Skeletal muscle biopsy tissue from family members (II.1, II.2, and II.4) with the *CCDC78* variant reveals aggregates of CCDC78 that localize to muscle cores marked by phalloidin enrichment (arrows). Scale bars = 30 µm.

Of the 21 VUS identified, a variant in the *CCDC78* gene (c.712A>C; p.Lys238Gln) was a strong candidate (Figure 7A,B, genotypes shown in Figure 1 and Table 1). A splice site variant in *CCDC78* resulting in an in-frame 74-amino-acid insertion has been identified in a family and linked to a dominant congenital myopathy with cores [74]. Affected individuals possessing this splice variant possessed muscle cores that colocalized with an abnormal accumulation of the CCDC78 protein and ryanodine receptor 1 (RyR1) [74]. By contrast, the *CCDC78* missense variant identified in the study presented here is relatively common in the population (0.0111 allele frequency, gnomAD v4.0.0, Broad Institute, accessed 14 November 2023) and, therefore, is unlikely to be singularly pathogenic. However, it is possible that the presence of the *LMNA* mutation sensitizes muscle to this variant in *CCDC78*, giving rise to profound skeletal muscle disease.

### 2.6. Individuals with the CCDC78 Variant Show Mislocalization of CCDC78

To determine if the *CCDC78* missense variant altered localization of the CCDC78 protein, skeletal muscle tissue from affected family members was stained with an antibody to CCDC78 and phalloidin (Table S1). Tissue from control individuals showed CCDC78 localization at the membrane of the muscle fibers and a focus of staining near the nuclear periphery, consistent with published reports of localization of wild-type CCDC78 in muscle [74] (Figure 7C). Similarly, skeletal muscle tissue from individual II.3, who possesses the *LMNA* mutation but lacks the *CCDC78* variant and does not have muscle cores, showed a wild-type localization pattern for CCDC78 (Figure 7C). By contrast, individuals II.1, II.2, and II.4, who possess both the *LMNA* mutation and the *CCDC78* variant and exhibit muscle cores, showed a dramatically different staining pattern. In these individuals, an antibody to CCDC78 stained cytoplasmic cores marked with phalloidin enrichment, suggestive of CCDC78 aggregation (Figure 7D). This abnormal pattern of staining was apparent in a minimum of 8% of muscle fibers in muscle biopsy cross sections (141 out of 1739 fibers, evaluated in individual II.4). Clear visualization of the CCDC78 enrichment was also observed in muscle costained with antibodies to CCDC78 and dystrophin (Table S1), which marks the periphery of the muscle fibers without obscuring the muscle cores (Figure S3).

### 2.7. CCDC78 Aggregates Colocalize with RYR1 Aggregates in Muscle Cores

**Figure 8:**
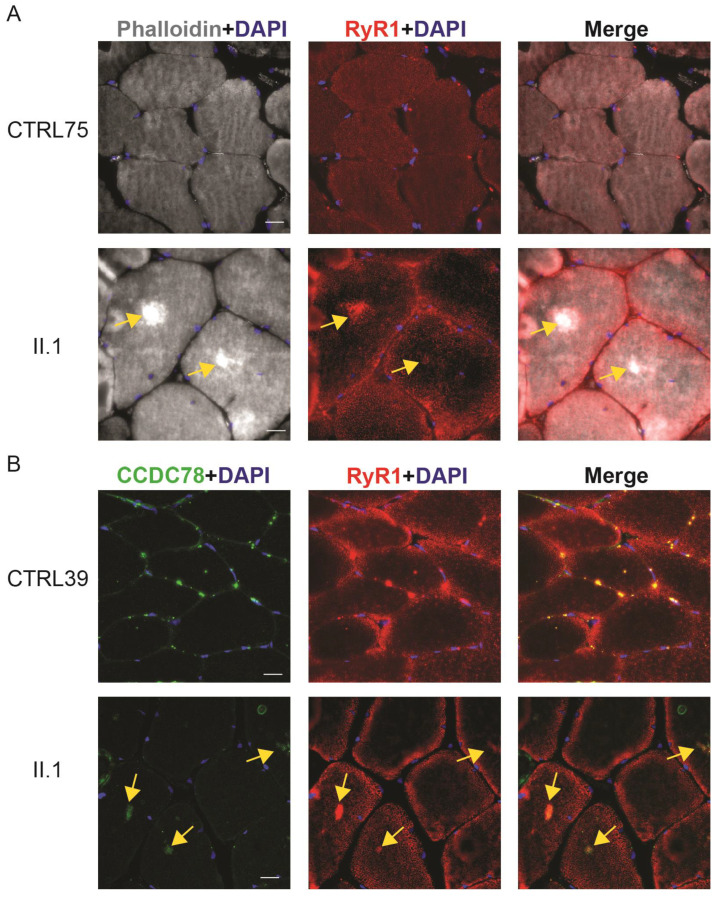
RyR1 also accumulates and colocalizes with CCDC78 in muscle cores of individuals with the CCDC78 variant. (**A**) Skeletal muscle biopsy tissue from individual II.1 stained with antibodies against phalloidin and RyR1 depicts accumulation of RyR1 localized in muscle cores indicated by the yellow arrows that are absent in an unrelated control (CTRL75). Scale bar = 30 µm. (**B**) Skeletal muscle biopsy tissue from individual II.1 stained with antibodies against CCDC78 and RyR1 reveals colocalization of CCDC78 with the RyR1 in muscle cores indicated by the yellow arrows. There are no cores and no colocalization of CCDC78 and RyR1 in an unrelated control (CTRL39). Scale bar = 30 µm.

In *CCDC78*-associated congenital myopathy, muscle cores that show aggregates of CCDC78 also contain aggregates of RyR1 [74]. To determine if the muscle biopsies from the individuals with the *CCDC78* variant also have aggregates of RyR1, we stained muscle cryosections with antibodies to RyR1 and phalloidin (Table S1). Muscle biopsy tissue from individual II.1 showed the presence of muscle cores as marked by areas of discoloration in the phalloidin staining and intense staining of RyR1 antibodies in these cores (Figure 8A). These features were absent in control muscle tissue (Figure 8A). A double stain of muscle tissue with antibodies against RyR1 and CCDC78 confirmed colocalization of these two proteins in muscle tissue from individual II.1 (see signal overlap in Figure 8B). These antibodies did not show overlap in an unrelated control (Figure 8B). Additionally, muscle biopsy tissue from individual II.3 also showed wild-type localization of CCDC78 and RyR1, demonstrating the specificity of RyR1 accumulation for those family members possessing muscle cores and the *CCDC78* variant (Figure S4). *RYR1* mutations are the most common cause of core myopathy [50,74]. It is important to note that we did not find *RYR1* DNA sequence variants in the family members in this study who underwent WGS. This indicates that mislocalization of RyR1 observed in the muscles of these individuals with the *LMNA* mutation and *CCDC78* variant is not due to a pathogenic variant in *RYR1*, indicating that the *CCDC78* variant is a likely cause of the muscle core pathology.

To determine if the muscle cores were a “sink” for misfolded proteins, we stained muscle tissue from individual II.1 with phalloidin and antibodies to the autophagy chaperone protein p62 that binds unfolded proteins and protein aggregates [75,76,77]. The phalloidin staining showed the characteristic marking of muscle cores; however, p62 did not colocalize with these cores, demonstrating that muscle cores are not generalized sinks for accumulated and likely aggregated proteins (Figure S5). Taken together, these data support that the *CCDC78* variant is causal for CCDC78 protein mislocalization and muscle core formation. 

**Figure 9:**
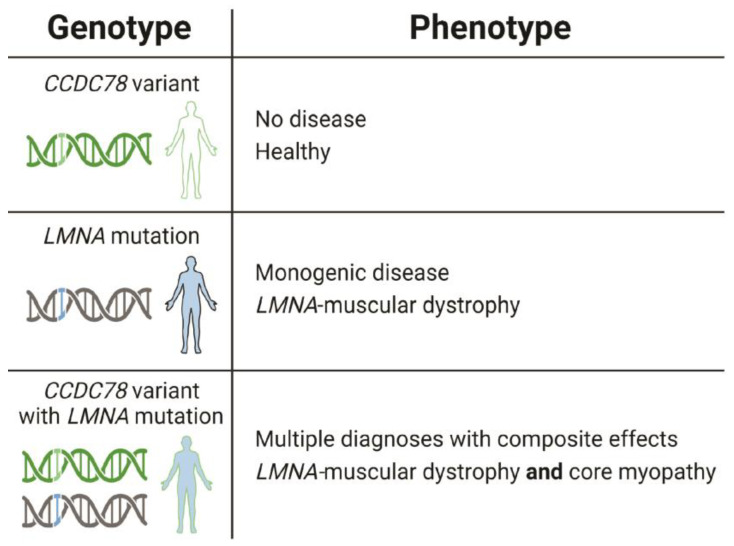
Summary of the genotype–phenotype relationship between *LMNA* and *CCDC78*. Individuals in the broader population with only the relatively common *CCDC78* variant are healthy. Individuals with the *LMNA* mutation present in this family have monogenic LGMD1B. When the *CCDC78* variant exists in combination with the *LMNA* mutation, the *LMNA* mutation sensitizes the muscle to the deleterious effects of the *CCDC78* variant, leading to core myopathy in addition to LGMD1B.

Collectively, our data indicate that a subset of individuals in this family diagnosed with LGMD1B due to a mutation in *LMNA* also have profound muscle weakness and wasting associated with muscle core pathology likely caused by a relatively common variant in *CCDC78*. Muscle cores are a rare phenotype for *LMNA*-associated muscle disease, strongly suggesting that these individuals have two separate muscle diseases, LGMD1B and core myopathy. The *LMNA* mutation appears to sensitize the muscle such that the *CCDC78* variant is sufficient to cause muscle cores that accumulate CCDC78 and RyR1 (Figure 9). Our findings provide an example of how individuals with *LMNA* mutations can exhibit phenotypic variation in muscle disease due to genetic background differences, even among closely related family members.

## 3. Discussion

We identified a four-generation family with LGMD and cardiomyopathy that was found to possess an *LMNA* mutation (Figure 1). Individuals in this family present with either skeletal muscle disease, cardiac disease, or both, an established phenotypic pattern of *LMNA*-associated striated muscle disease [14,15,16,19,23,30,31,32,35,39,42]. The *LMNA* mutation present in this family is c.639+1G>A, which activates a cryptic splice site between exon three and four, resulting in a frame shift at amino acid 212 and the introduction of a premature stop codon (Figure 5). In addition to the *LMNA* mutation, a subset of family members has a relatively common *CCDC78* variant. These family members exhibit a more profound skeletal muscle disease than the family members with only the *LMNA* mutation (Figure 7). Thus, the pathogenic *LMNA* mutation appears to sensitize the muscle to the *CCDC78* variant such that individuals with both variants develop two types of muscle disease (Figure 9).

Most *LMNA* mutations that cause muscle disease result in single amino acid substitutions throughout the length of the lamin A/C protein [12,16,17,23]. While pathogenic splice and nonsense mutations in *LMNA* have been identified [78,79,80,81,82,83,84,85,86,87], they are generally less common than missense mutations [11,12,13,16,17,23,31,35,37,42,88,89,90,91]. The disease mechanisms of these splice and nonsense mutations remain poorly understood. Missense mutations in *LMNA* are often considered to be dominant negatives in which the mutant lamins incorporate into the lamina and disrupt the lamin network, resulting in abnormally shaped nuclei [12,13,88,89,90,91]. By contrast, splice or nonsense mutations resulting in a premature stop codon are expected to be functional nulls and result in haploinsufficiency [78,79,80,81,82,83,84,85,86,87]. However, reports of haploinsufficiency in families with these mutations vary, with some reports showing no change in lamin A/C mRNA and protein levels [80,81,83,85]. In these cases, it is possible that there is upregulation of the wild-type *LMNA* allele, or differences in protein levels exist between the differentiated muscle cells, which would not be evident from western analysis of protein extract from whole muscle tissue [80,81,83,85]. Thus, additional research is required to understand the mechanisms by which splicing defects and nonsense mutations in *LMNA* cause muscle pathology. 

The *LMNA* splicing mutation sensitizes the muscle physiology such that the relatively common variant in *CCDC78* leads to muscle core formation. The presence of muscle cores as part of the pathology of *LMNA*-MD is highly atypical, with only one published report showing skeletal muscle biopsy tissue from an individual with an *LMNA* mutation possessing muscle cores [92]. Muscle cores are also not typically part of the pathology of LGMD [28], making the presence of cores a unique finding in this family.

Core myopathies represent a heterogeneous collection of diseases united by the presence of regions within individual muscle fibers lacking oxidative enzyme activity associated with a paucity of mitochondria and with varying degrees of ultrastructural sarcomeric disruption known as muscle cores [50,51,52,59,60]. Core myopathies can be further subdivided into different histopathologic morphologies termed central core disease, minicore or multiminicore myopathy, dusty core disease, and core rod myopathy, with central core disease being the most common [50,51,52]. Most cases of central core disease are caused by mutations in *RYR1* [52,61,93]. However, the genetic cause of core myopathies overall is extremely heterogeneous, with at least 15 genes implicated [50]. Overall, the nature of cores has not been well characterized aside from the paucity of mitochondria and ultrastructural sarcomeric disruption within the muscle fibers. More research is required to better understand the mechanisms and pathophysiology of core formation, which is also currently not well understood [50,51]. 

A link between *CCDC78* and core myopathy has been previously reported [74], and *CCDC78* is included as a core myopathy gene in review publications [50,94]. Little is known about the endogenous functions of CCDC78. In Xenopus, CCDC78 is a deuterosome-associated protein that is required for centriole amplification [95,96,97]. In skeletal muscle, CCDC78 localizes to the sarcolemma, perinuclear region, and the triad [74], the structural interface between the T-tubule and portions of the sarcoplasmic reticulum [94]. Given that coiled-coil domain-containing proteins are known to play a role in oligomerization of large protein complexes [98,99], it is possible that CCDC78 is necessary for the structural integrity of the triad [94]. We and others have demonstrated that myopathy-associated variants in *CCDC78* lead to the mislocalization of RyR1 [74], a calcium channel protein that localizes to the triad [94]. Mislocalization of RyR1 suggests that disruptions in calcium homeostasis and excitation–contraction coupling might be exacerbating factors in individuals in this family with both the *LMNA* mutation and *CCDC78* variant [94]. A direct link between CCDC78 and lamins remains undefined. Whether the DNA variant in *CCDC78* and the mutation in *LMNA* interact genetically or cause separate insults to muscle function requires further investigation. 

Whole genome sequencing of six individuals in the family investigated here determined that individuals II.1, II.2, II.4, and III.2 possess the *CCDC78* variant, while individuals II.3 and III.5 do not. Further analysis with Sanger sequencing revealed that the *CCDC78* variant was not transmitted from individual I.1, suggesting that I.2 is a carrier of the *CCDC78* variant. It is likely that individual I.2 did not exhibit skeletal muscle disease because they died young, prior to onset of muscle weakness (Figure 1). We report segregation of the *CCDC78* variant only in individuals with muscle core pathology among the four generation II siblings. Individual III.2 also has the variant but has not had a muscle biopsy to confirm the presence of cores. He had an unusual phenotype of mild muscle weakness in childhood that resolved and had normal muscle function in young adulthood. Given the presence of the *CCDC78* variant in this individual and their earlier symptoms, it is possible that this individual will develop symptomatic weakness, likely in the next decade.

The *CCDC78* variant present in this family is c.712A>C, which is in the coding region of exon 8 of the gene and is predicted to cause the amino acid substitution p.Lys238Gln. This variant is relatively common in the general population, with an allele frequency of 0.0111 and 120 homozygotes reported in the gnomAD database (Broad Institute, accessed 21 November 2023, v4.0.0). Because of the prevalence of this variant, it is unlikely that this variant is individually pathogenic, as the prevalence of the variant far outweighs the prevalence of core myopathy. However, it is possible that the deleterious effect of this variant is enhanced by the rare *LMNA* mutation that already compromises muscle function in a subset of family members.

In conclusion, our data suggest that the skeletal muscle abnormalities in this family represent two diseases caused by two distinct DNA sequence changes in two different genes. The rare *LMNA* mutation in this family causes LMGD1B; however, it does not account for the muscle core pathology. The relatively common *CCDC78* in this family segregates with core myopathy and the more profound skeletal muscle weakness and wasting (Figure 9). Our results provide additional insight into the variability associated with *LMNA*-MD by supporting the hypothesis that the wide phenotypic spectrum of *LMNA*-MD is at least in part driven by DNA sequence variants in the genetic background of affected individuals [16,42,43,44,45]. Identification of these additional genetic variants that contribute to the skeletal muscle pathology can aid in diagnosis and prognostic predictions, be used to stratify individuals in clinical trials, and offer new potential targets for therapy.

## 4. Materials and Methods

### 4.1. Patient Recruitment

Family members were identified through the University of Iowa Neuromuscular Multispecialty Clinic and Heart and Vascular Center. Institutional Review Board approval (ID#200510769) was obtained through the University of Iowa. Medical histories were abstracted from the electronic health record.

### 4.2. Tissue Collection and Analysis

Clinical diagnostic muscle biopsies were processed in a routine fashion for evaluation of cryosections and for embedment in either paraffin or epon blocks. Cryosections were stained with hematoxylin and eosin (H&E) and cytochrome C oxidase (COX). They were also stained with antibodies that evaluate muscle diseases, including dystrophin and embryonic myosin heavy chain (Table S1) [53]. Epon-embedded muscle was evaluated as 1 µm-thick sections stained with toluidine blue and imaged with electron microscopy. Both the autopsied and explanted hearts were grossly evaluated in the standard fashion and fixed in formalin. Selected regions of the hearts were embedded in paraffin blocks and evaluated with H&E and Masson trichrome stains.

Skin biopsies were obtained on a research basis from subjects II.1, II.2, and III.5, and fibroblasts were established using a research protocol approved by the University of Iowa Institutional Review Board for the Wellstone Muscular Dystrophy Specialized Research Center (IRB ID#200510769), expanded by passaging in culture flasks, and cryopreserved. The growing cells were maintained at 37 °C and 5% CO_2_ in DMEM medium plus 20% fetal bovine serum and 0.5% penicillin–streptomycin (Invitrogen, Carlsbad, CA, USA). Cells in cryovials were stored in liquid N_2_.

### 4.3. Identification of the LMNA Variant

The sequence of the *LMNA* gene was evaluated with traditional Sanger sequencing of genomic DNA extracted from peripheral blood leukocytes of family members. The 12 coding exons and the flanking intronic boundaries were amplified with PCR and sequenced in both directions. The patient DNA sequence was compared to the *LMNA* reference sequence (NM_170707). 

### 4.4. In Silico Transcript Analysis

We employed NNSplice, SpliceScan I, NetGene2, GeneSplicer, and MaxEntScan [56,100,101,102,103,104,105,106,107,108], five freely available and frequently used online in silico computational tools, to predict whether the *LMNA* mutation under study would likely alter the normal pre-mRNA splicing of *LMNA* [56,100,101,102,103,104,105,106,107,108]. The results obtained were then integrated using GENSCAN (Massachusetts Institute of Technology, Cambridge, MA, USA), SpliceScan II (New Mexico State University, Las Cruces, NM, USA), and ExonScan (Massachusetts Institute of Technology, Cambridge, MA, USA) to predict how the mis-spliced mRNA transcript may differ from the wild-type mRNA [109,110,111,112]. Twenty percent or greater change in the splice site strength/score was considered significant and likely to alter pre-mRNA splicing of the *LMNA* message [113,114].

### 4.5. RNA Isolation and RT-PCR Analysis

RNA was extracted from fibroblast cultures (~70–80% confluent) according to the manufacturer’s instructions (TriReagent; Molecular Research Center, Cincinnati, OH, USA and TransMax Nuclear Extraction Kit; Genlantis, San Diego, CA, USA). 

cDNA synthesis from the isolated RNA was carried out using oligo dT primers included in the SuperScript III First-Strand Synthesis System (Invitrogen catalogue number 18080-051, Carlsbad, CA, USA). RT-PCR analysis was carried out using 2.5× Eppendorf HotMasterMix (Eppendorf, Hamburg, Germany) according to the manufacturer’s instructions. RT-PCR primers were designed to span exon:exon junctions to prevent amplification of genomic DNA. RT-PCR reaction products were analyzed with 1–2% agarose gel electrophoresis. Primer sequences used for these reactions are available in Table S2.

Sequencing of PCR products amplified from peripheral blood leukocytes and fibroblast-derived cDNA was performed at the University of Iowa Institute of Human Genetics-Genomics Division Core facility following purification with the QIAquick PCR Purification Kit (Qiagen, Germantown, MD, USA).

### 4.6. Whole Genome Sequencing and Bioinformatic Analysis

DNA extracted from peripheral blood leukocytes for clinical use was obtained from the University of Iowa Hospital and Clinics (UIHC) Molecular Pathology Laboratory with Institutional Review Board approval (IRB#200510769) and treated with RNase H. After treatment, genomic DNA was concentrated using the Zymo Research Genomic DNA Clean & Concentrator Kit (Zymo Research, catalog #D-4010, Irvine, CA, USA) and the amount of DNA was measured using a NanoDrop 2000 (Thermo Scientific, Waltham, MA, USA). DNA samples were electrophoresed on a 1% agarose/TAE gel to ensure that the gDNA was not degraded and concentration was measured again with a Qubit dsDNA HS Assay Kit (Molecular Probes/Life Technologies, catalog #Q32851, Carlsbad, CA, USA) and a Qubit 2.0 Fluorometer to ensure high quality for sequencing. Samples were submitted to the Iowa Institute of Human Genetics-Genomics Division Core facility for whole genome sequencing. Samples were sequenced using an Illumina NovaSeq 6000 with an S4 flow cell. Sequencing was run using paired end reads for 300 cycles for a mean depth of coverage of 35X.

Reads were aligned to the GRCh37/UCSC hg19 reference genome with BWA-MEM, a Burrows–Wheeler aligner, and results were stored in the SAM/BAM format [62]. Single-nucleotide variants and small insertions/deletions were detected using HaplotypeCaller from the Genome Analysis Toolkit (Broad Institute, Cambridge, MA, USA) [115]. Variants were then annotated and filtered in VarSeq for quality control, proper phenotype segregation, a minor allele frequency of <5% in the gnomAD and 1000 Genomes databases [73,116], and to predict loss-of-function or missense mutations (VarSeq [Version 2.3]; Golden Helix, Inc., Bozeman, MT, USA). Retained variants and the genes containing them were then prioritized and ranked using a combination of conservation metrics (GERP, PhyloP), constraint metrics (missense z-score and pLI), in silico predictors of pathogenicity (SIFT, PolyPhen-2, MutationTaster, MutationAssessor, FATHMM, and FATHMM-MKL), relevant tissue expression (GTEx), and PhoRank, a tool available in VarSeq modeled on the Phevor algorithm, that combines phenotype, gene function, and disease information from many ontologies, to prioritize disease-causing alleles [62,63,64,65,66,67,68,69,70,71,72,73].

### 4.7. Immunostaining of Skeletal Muscle Tissue

Residual frozen tissue from diagnostic human muscle biopsies were obtained from the University of Iowa Wellstone Muscular Dystrophy Specialized Research Center. Cryosections were prepared on glass slides and maintained at −80 °C. Immunostaining was preformed according to a previously published protocol [90]. In brief, slides were placed in blocking buffer [PBS2+ (130 mM NaCl, 3 mM Na_2_HPO_4_, 3 mM NaH_2_PO_4_, 10 mM EGTA), 0.1% Triton-X100, 0.1% BSA] for 1 h at room temperature. Primary antibodies were diluted in blocking buffer and 40 microliters of primary antibody solution were pipetted onto each sample. Samples were covered with parafilm and incubated in the dark in a sealed humidified chamber for one hour at room temperature. Slides were washed in PBS2+ and stained with appropriate secondary antibodies conjugated to Alexa-488, Rhodamine Red-X, or Texas Red-X (Invitrogen, Thermo Fisher Scientific, Waltham, MA, USA) and under the same conditions as the primary antibody. Slides were washed with PBS2+ and a coverslip mounted with 10 microliters of Vectashield with DAPI (Vector Laboratories, Newark, CA, USA). Slides were imaged on a Leica THUNDER Imager Live Cell & 3D Assay imaging system using a 63X objective (Leica Microsystems, Wetzlar, Germany).

## Figures and Tables

**Table 1:** Clinical summaries of family members with the *LMNA* c.639+1G>A variant.

Patient ID (Age at Last Evaluation)	Sex	Age at Onset of Symptomatic Muscle Weakness (Years)	Age at Fulltime Wheelchair Current Motor Ability (Years)	Age at First Abnormal Echocardiogram (Years)	Age of ICD Placement (Years)	CK (U/L) ^2^	Skeletal Muscle Pathology (Age at Biopsy; Years)	*CCDC78* c.712A>C Variant
I.2 (deceased at age 31)	Female	N/A	N/A	27	Not Done	>600	Not evaluated	Not tested ^5^
II.1 (60 years)	Female	mid 20s	59	Normal echocardiogram at 59	50	617	Mildly dystrophic with cores (34)	Present
II.2 (62 years)	Male	45	Able to walk short distances at 60	On initial evaluation at 41 ^1^	Not Done ^1^	1000	Mildly dystrophic with cores (29) ^3^	Present
II.3 (60 years, deceased at age 60)	Male	50–55	Ambulatory at age 60 with nearly normal strength	47 ^4^	48	281	Type II fiber predominance, rare necrotic fibers (47) ^3^	Absent
II.4 (65 years)	Female	~30	60	68	58	610	Mildly dystrophic with cores (31)	Present
III.1 (28 years)	Male	N/A	No weakness at 28	Normal cMRI at 28	28, for symptomatic arrhythmia	N/A	No biopsy	Not tested
III.2 (32 years)	Male	N/A	No weakness at 32	Normal echocardiogram at 32	N/A	N/A	No biopsy	Not tested
III.5 (30 years)	Male	N/A	No weakness at 31	29	30	288	No biopsy	Present
III.6 (38 years)	Male	N/A	No weakness at 38	Normal cMRI at 38	N/A	813	No biopsy	Not tested

^1^: Heart transplantation at age 43. ^2^: Earliest recorded value. ^3^: Conducted because of family history of muscle weakness, patient had normal strength at the time of biopsy. ^4^: LVEF decreased to 35–40% upon initial evaluation, onset age presumed to be earlier. ^5^: Samples from this individual were not available for testing; however, it is likely that they possess the *CCDC78* variant given that it is absent in individual I.1. Individual I.2 died before developing skeletal muscle weakness.

**Table 2:** Predicted effects of DNA sequence variants identified via whole genome sequencing.

Gene Symbol	Mutation Type	Transcript Consequence	Protein Consequence	gnmoAD Allele Frequency ^1^
*CCDC78*	Missense	c.712A>C	p.Lys238Gln	1.11 × 0^−2^
*VPS13A*	Splice region	c.385+6_385+15delGAAAACAGTA	Potential splicing effect	5.39 × 10^−3^
*AXIN1*	Missense	c.1948G>A	p.Gly650Ser	1.87 × 10^−2^
*TMC4*	Stop retained	c.2120G>A	p.Ter707=	1.58 × 10^−2^
*PTPRD*	Missense	c.2341A>G	p.Thr781Ala	2.74 × 10^−2^
*TDO2*	Missense	c.685A>C	p.Asn229His	3.87 × 10^−2^
*SPIN1*	Splice region	c.590-8delT	Potential splicing effect	2.39 × 10^−1^
*SPIN1*	Splice region	c.590-9_590-8delTT	Potential splicing effect	1.26 × 10^−1^
*ZNF343*	Missense	c.1373C>T	p.Thr458Met	5.65 × 10^−3^
*WDR90*	Splice region	c.1380-8G>A	Potential splicing effect	3.36 × 10^−2^
*FNIP2*	Missense	c.1615G>C	p.Gly539Arg	1.02 × 10^−2^
*WNK2*	In-frame deletion	c.4190_4204delATGGAGCAGCTCCAG	p.Asp1397_Pro1401del	2.56 × 10^−4^
*KIF27*	Missense	c.2579G>A	p.Arg860Gln	3.50 × 10^−3^
*RNF151*	Splice region	c.149+8C>T	Potential splicing effect	4.71 × 10^−3^
*ZNF75A*	Premature start codon gain	c.540C>T	p.Tyr180=	1.16 × 10^−2^
*RPL3L*	Missense	c.784G>A	p.Val262Met	2.86 × 10^−2^
*SIRPD*	Missense	c.206G>A	p.Gly69Glu	9.42 × 10^−5^
*HEXD*	Missense	c.1299C>T	p.Ala433=	Not reported
*JMJD8*	Splice region	c.323-6C>T	Potential splicing effect	3.36 × 10^−2^
*PRR35*	Missense	c.1504G>A	p.Gly502Ser	3.03 × 10^−2^
*TUT7*	Missense	c.1679G>A	p.Arg560Gln	4.80 × 10^−3^

^1^, Accessed on 4 April 2024, gnomAD v4.0.0.

## Data Availability

The raw data supporting the conclusions of this article will be made available from the authors on request.

## Supplementary Materials

The following supporting information can be downloaded at: https://www.mdpi.com/article/10.3390/ijms25094930/s1.
