# Supplementary material for: The Influence of a Genetic Variant in CCDC78 on LMNA-Associated Skeletal Muscle Disease
Source: Int J Mol Sci. 2024 Apr 30;25(9):4930. doi: 10.3390/ijms25094930 (PMC11084688; doi:10.3390/ijms25094930)
Supplement: Supplementary file 1 [file ijms-25-04930-s001.zip › ijms-2939238-supplementary.pdf]

# The influence of a genetic variant in *CCDC78* on *LMNA*-associated skeletal muscle disease

**Nathaniel P. Mohar [1,2], Efrem M. Cox [3,4], Emily Adelizzi [1,5], Steven A. Moore [3], Katherine D. Mathews [6], Benjamin W. Darbro *[1,6] and Lori L. Wallrath *[1,2]**

## Supplementary materials

**Table S1.** Antibody List.

| Antigen | Use | Antibody Type | Source | Product Number | Dilution |
|---|---|---|---|---|---|
| Merosin | IHC | Rat monoclonal | Millipore (clone 4H8) | MAB1922 | 1:100 |
| Embryonic myosin heavy chain (eMHC) | IHC | Mouse monoclonal | Developmental Studies Hybridoma Bank (DSHB) | F1.652 | 1:100 |
| Lamin A/C (N18) | IHC | Goat polyclonal | Santa Cruz Biotechnology, Inc. | Sc-6215 | 1:200 |
| Emerin (H12) | IHC | Mouse monoclonal | Santa Cruz Biotechnology, Inc. | Sc-25284 | 1:500 |
| FG-repeat containing nuclear pore proteins (MAB414) | IHC | Mouse monoclonal | Covance | E11CF00696 | 1:2,000 |
| CCDC78 | IHC | Rabbit polyclonal | ThermoFisher | PA5-44001 | 1:100 |
| Texas Red-X Phalloidin | IHC | N/A | Invitrogen Molecular Probes | T7471 | 1:400 |
| Dystrophin | IHC | Mouse monoclonal clone 6A9 | Developmental Studies Hybridoma Bank | MANEX50 (6A9) | 1:100 |
| P62/SQSTM1 | IHC | Mouse monoclonal | R&D Systems | MAB8028-SB | 1:200 |
| RyR1 | IHC | Mouse monoclonal | Developmental Studies Hybridoma Bank | 34C | 1:100 |

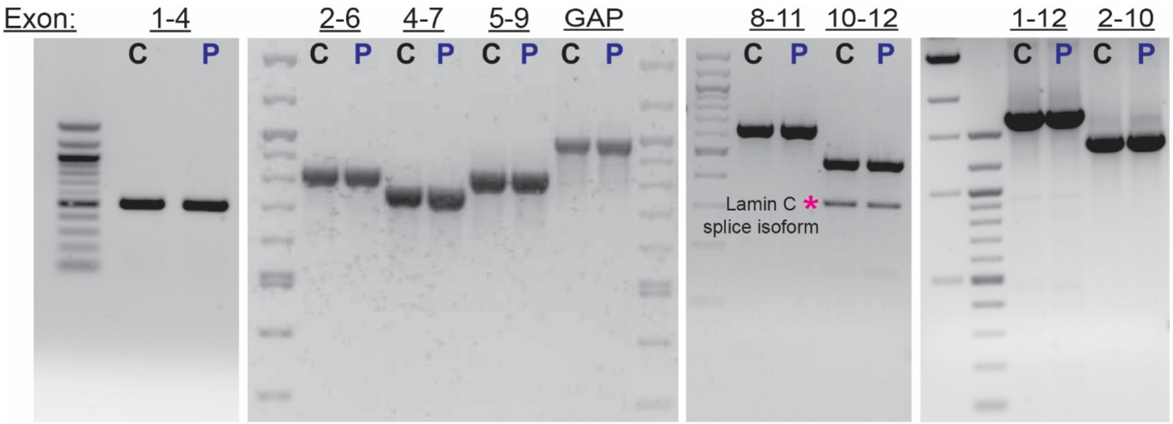


**Figure S1.** RT-PCR analysis of *LMNA* transcripts did not detect grossly abnormally sized products. RT-PCR analysis was performed on total mRNA extracted from control fibroblasts (C) and fibroblasts taken from an affected family member (P, II.2). All twelve exons of *LMNA* were interrogated in a stepwise fashion with over-lapping amplicons spanning exons 1-4, 2-6, 4-7, 5-9, 8-11, and 10-12. Full length *LMNA* cDNA was also amplified (exons 1-12) as well as the cDNA that produces the lamin C protein isoform (2-10). PCR amplicons were all of the appropriate size and no differentially expressed products were detected between the control and affected fibroblasts.

**Table S2.** Primer sequences used for RT-PCR amplification of the *LMNA* gene mRNA transcript and anticipated amplicon sizes.

| Exons Amplified | Forward Primer | Reverse Primer | Anticipated Product |
|---|---|---|---|
| Exons 1-4 | GCTGGAGCTGAG-CAAAGTGCGTGAGG[1] | GTCCAGCTTGGCAGAATAAGTC | 508bp |
| Exons 2-3 | AAGCGCGCAATACCAAGAAG | CTTGGTCTCACGCAGCTCT[2] | 308bp |
| Exons 2-6 | AAGCGCGCAATACCAAGAAG | GACAGGCGTAGCCTCTCCTC | 820bp |
| Exons 4-7 | AGTGAGGAGCTGCGTGAGAC | ACTGGTCCTCATTGGACTTGTT | 754bp |
| Exons 5-9 | AGAAGACTTATTCTGCCAAGCTG | ACTTCTTCCCCAGTGGAGTTGAT | 823bp |
| Exons 8-11 | CAACAAGTCCAATGAGGACCAG | AGGTCCCAGATTACATGATGCT | 641bp |
| Exons 10-12 | ATCAACTCCACTGGGGAAGAAG | GGCATGAGGTGAGGAGGAC | 466bp |
| Exons 1-12 | GCTGGAGCTGAG-CAAAGTGCGTGAGG[1] | ACATGATGCTGCAGTTCTGG[1] | 1685bp |
| Exons 2-10 (Lamin C) | AAGCGCGCAATACCAAGAAG | CGGCTACCACTCACGTGGTG[3] | 1360bp |
| GAPDH[4] | TGAAGGTCGGAGTCAACGGATTTGGTCATGTGGGCCATGACCTCCACCAC | | 983bp |

[1]: Primer does not cross an exon:exon junction. All remaining primers do span an exon:exon junction (for example, the forward primer for amplification of exons 2-6 spans the exon 1:exon 2 junction).

[2]: Underlined nucleotide is a mismatch to the wildtype spliced mRNA transcript at the exon 3:exon 4 junction, however, is a match to a proposed mutation induced abnormally spliced mRNA product predicted by *in silico* analysis to utilize an upstream 5′ cryptic donor splice site.

[3]: Reverse primer is specific for a portion of exon 10 differentially spliced and present only in the mRNA transcript that gives rise to the lamin C protein isoform.

[4]: GAPDH was amplified as an internal amplification control

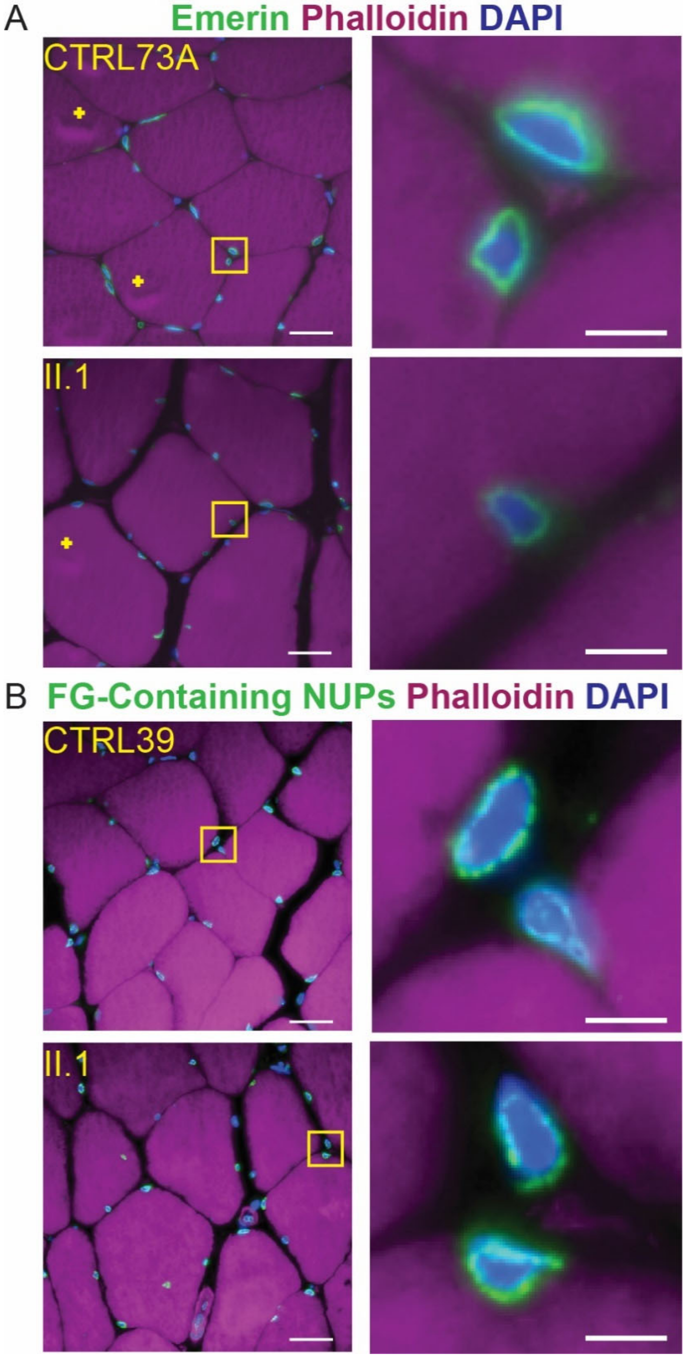


**Figure S2: Emerin and nuclear pore proteins are properly localized in individuals with the *LMNA* mutation.** A) Skeletal muscle biopsy from individual II.1 stained with an antibody against emerin, an inner nuclear envelope protein, reveals nuclear envelope localization, like that of the control (CTRL73A). Scale Bar = 30 μm. B) Skeletal muscle biopsy from individual II.1 stained with an antibody against FG repeat-containing nuclear pore proteins reveals nuclear envelope localization, like that of the control (CTRL39). Scale Bar = 30 μm.

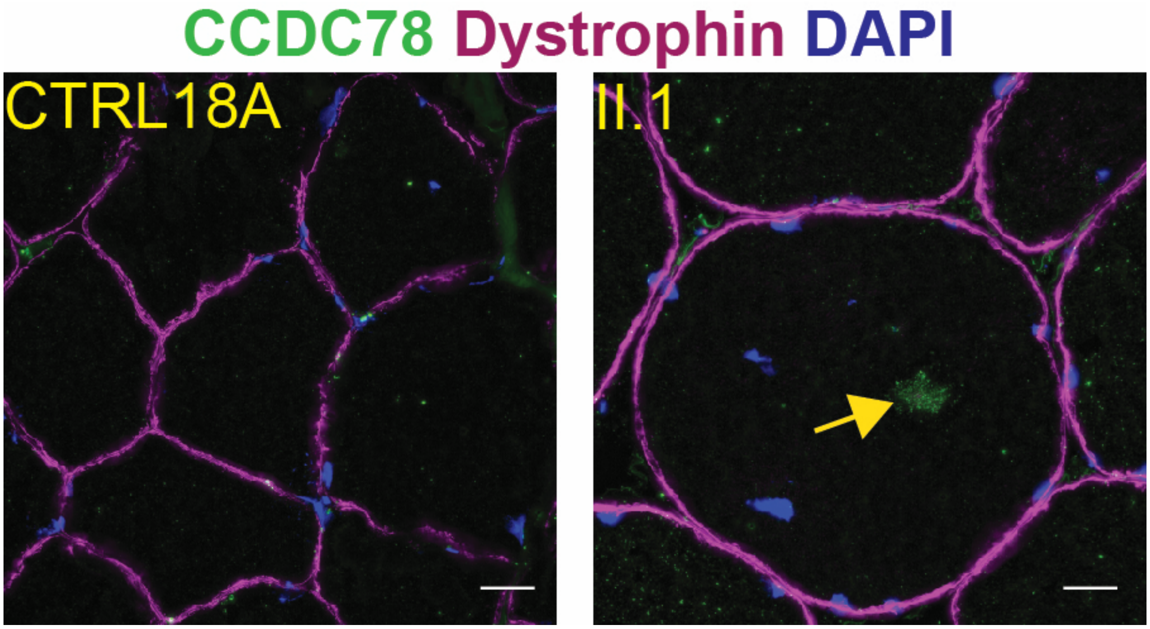


**Figure S3.** CCDC78 accumulation in the cytoplasm is obvious when the muscle cell periphery is marked with antibodies to dystrophin. Skeletal muscle biopsy tissue from individual II.1 stained with antibodies against CCDC78 and dystrophin shows obvious CCDC78 accumulation within the cytoplasm that is outlined by the dystrophin staining just under the sarcolemmal membrane. This demonstrates that the cytoplasmic CCDC78 staining in Figures 7 and 8 is not due to bleed through from fluorescent signal from other channels. Cytoplasmic CCDC78 accumulation is absent in the muscle of an unrelated control (CTRL18A). Scale bar = 30 μm.

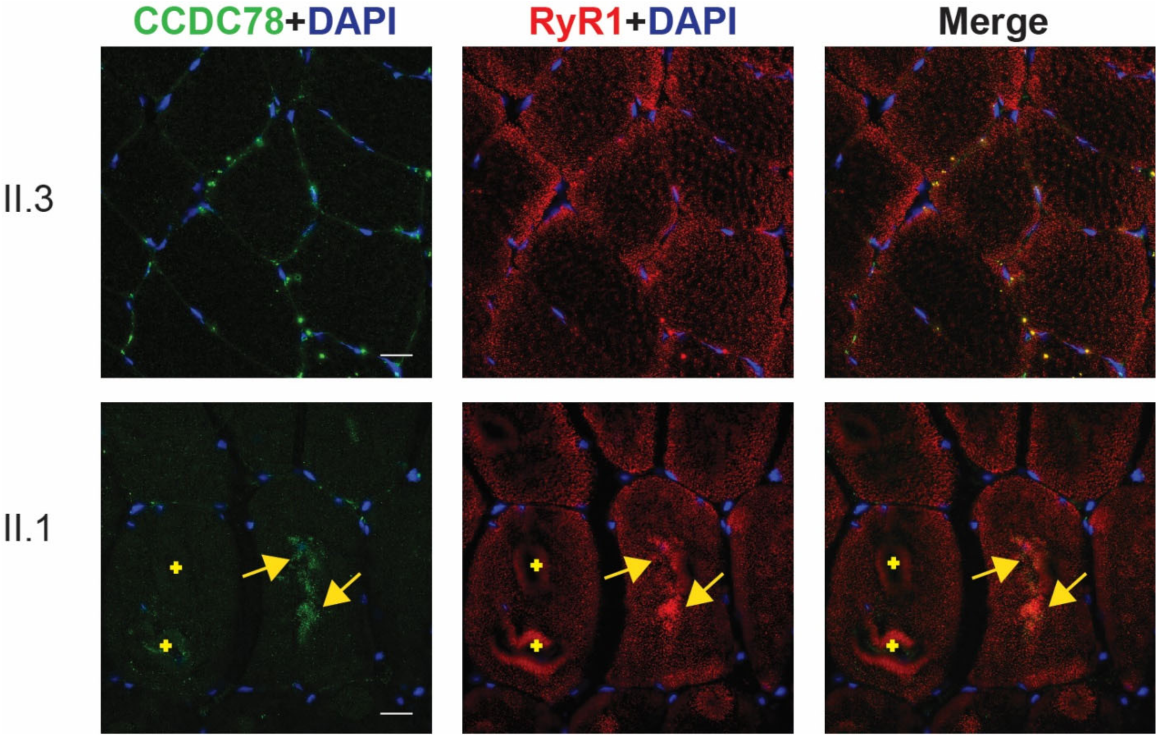


**Figure S4.** RyR1 accumulation is absent in individual II.3. Skeletal muscle biopsy from individual II.3, who possesses the *LMNA* mutation but lacks the *CCDC78* variant, stained with antibodies against CCDC78 and RyR1 show normal CCDC78 and RyR1 localization. In contrast, skeletal muscle biopsy from II.1, a sibling of II.3 who possesses both the *LMNA* and *CCDC78* variants, shows abnormal accumulation of both CCDC78 and RyR1 that colocalize within the cytoplasm. + = ice crystal artifacts from tissue preservation.

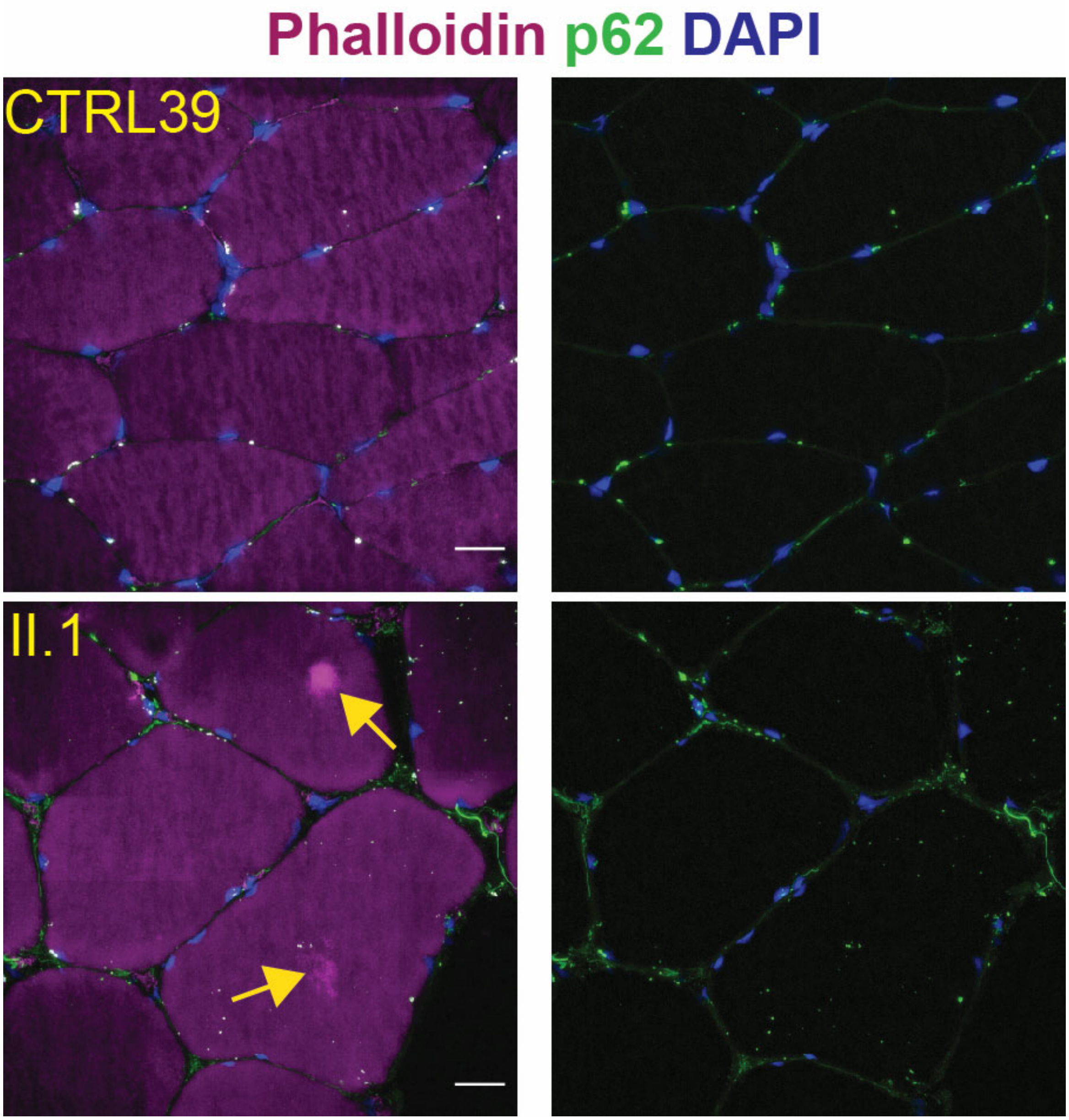


**Figure S5.** The muscle cores in profoundly affected family members are not marked by p62. Skeletal muscle biopsy from individual II.1 stained with an antibody against the p62 autophagy protein and phalloidin show muscle cores that lack staining with an antibody to p62. No cores and no p62 staining are observed in the control muscle (CTRL39). Scale bar = 30 µm.
